# Mutant p53 murine oviductal epithelial cells induce progression of high-grade serous carcinoma and are most sensitive to simvastatin therapy in vitro and in vivo

**DOI:** 10.1186/s13048-023-01307-x

**Published:** 2023-11-20

**Authors:** Madison Pereira, Alice Glogova, Jacob Haagsma, Julia Stewart, Trevor G. Shepherd, Jim Petrik

**Affiliations:** 1 Department of Biomedical Sciences, University of Guelph, Guelph, ON N1G 2W1 Canada; 2 The Mary & John Knight Translational Ovarian Cancer Research Unit, London Regional Cancer Program, London, ON Canada; 3 Department of Anatomy & Cell Biology, Schulich School of Medicine and Dentistry, Western University, London, ON Canada; 4 Department of Oncology, Schulich School of Medicine and Dentistry, Western University, London, ON Canada; 5 Department of Obstetrics & Gynaecology, Schulich School of Medicine and Dentistry, Western University, London, ON Canada

**Keywords:** Ovarian cancer, p53, Mevalonate pathway, Fallopian tube orthotopic model, Simvastatin

## Abstract

High-grade serous carcinoma (HGSC) is the most common and aggressive subtype of epithelial ovarian cancer, characterized by gain-of-function *TP53* mutations originating in the fallopian tube epithelium. Therapeutic intervention occurs at advanced metastatic disease, due to challenges in early-stage diagnosis, with common disease recurrence and therapy resistance despite initial therapy success. The mevalonate pathway is exploited by many cancers and is potently inhibited by statin drugs. Statins have shown anti-cancer activity in many, but not all cancers. Here, we investigated the role of p53 status in relation to mevalonate pathway signaling in murine oviductal epithelial (OVE) cells and identified OVE cell sensitivity to statin inhibition. We found that p53^R175H^ mutant and *Trp53* knockout OVE cells have increased mevalonate pathway signaling compared to p53 wild-type OVE cells. Through orthotopic implantation to replicate the fallopian tube origin of HGSC, p53^R175H^ mutant cells upregulated the mevalonate pathway to drive progression to advanced-stage ovarian cancer, and simvastatin treatment abrogated this effect. Additionally, simvastatin was more efficacious at inhibiting cell metabolic activity in OVE cells than atorvastatin, rosuvastatin and pravastatin. In vitro, simvastatin demonstrated potent effects on cell proliferation, apoptosis, invasion and migration in OVE cells regardless of p53 status. In vivo, simvastatin induced ovarian cancer disease regression through decreased primary ovarian tumor weight and increased apoptosis. Simvastatin also significantly increased cytoplasmic localization of HMG-CoA reductase in ovarian tumors. Downstream of the mevalonate pathway, simvastatin had no effect on YAP or small GTPase activity. This study suggests that simvastatin can induce anti-tumor effects and could be an important inhibitor of ovarian cancer progression.

**Supplementary Information:**

The online version contains supplementary material available at 10.1186/s13048-023-01307-x.

## Introduction

Ovarian cancer is the most lethal gynaecological cancer and fifth leading cause of cancer-related deaths in women, with a 5-year survival rate of less than 42% [1]. High-grade serous carcinomas (HGSCs) are the most common and aggressive subtype of ovarian cancer, with *TP53* mutations occurring in over 96% of HGSC cases [2]. HGSC was originally thought to arise within the ovarian surface epithelium, however, acquisition of gain-of-function *TP53* mutations in the distal fallopian tube epithelium (FTE) is now thought to be the initiating event. Within the FTE, a pre-malignant serous-tubal intraepithelial carcinoma (STIC) lesion is formed that eventually colonizes on the ovary to develop a primary ovarian tumor and progress to HGSC [3, 4].

The hallmarks of cancer encompass several adaptations that cancer cells undertake to facilitate their survival. One of these hallmarks involves the upregulation of metabolic pathways to facilitate rapid proliferation, uncontrollable growth, invasion and metastasis [5]. Previously, our lab discovered that metastatic ascites cells (28–2) from our orthotopic, syngeneic murine model of ovarian cancer were p53 mutant, compared to p53 wild-type ID8 cells used for tumor induction [6], suggesting interaction with the ovarian microenvironment initiated the development of a *TP53* mutation. The R175H gain of function p53 mutation has the highest occurrence across a spectrum of cancers, and is present in 80% of high-grad serous ovarian cancers [7, 8]. Sequencing of these metastatic 28–2 cells revealed majority of genes upregulated were from the mevalonate pathway [6]. This pathway is recognized for its role in cholesterol biosynthesis and protein prenylation, a key post-translational modification that facilitates activation of many signaling proteins, which is implicated in the progression of ovarian cancer [9–14].

HMG-CoA reductase (HMGCR), the rate-limiting enzyme of the mevalonate pathway, is potently inhibited by statin drugs. Epidemiological and retrospective studies demonstrated statins improve overall survival in cancer patients [15, 16], including those with ovarian cancer [17, 18]. In our ovarian cancer model, where metastatic cells had increased mevalonate signaling, treatment with simvastatin significantly reduced disease burden [6]. Interestingly, rescue experiments proved geranylgeranyl pyrophosphate and mevalonate could reverse simvastatin-induced apoptosis, however, cholesterol could not [6]. Therefore, simvastatin may target oncogenic downstream signaling, including small GTPases and the Hippo pathway.

Bridging these concepts, we investigated the relationship between oviductal epithelial (OVE) cell p53 status (wild-type, p53^R175H^ mutant or *Trp53* knockout) on mevalonate pathway expression, ovarian cancer disease progression and sensitivity to statin drugs. We anticipated p53^R175H^ mutant OVE cells to have the greatest mevalonate pathway expression, progress to advanced-stage ovarian cancer in vivo and be most sensitive to statin treatment.

## Materials and methods

### Cell lines and culture

Murine OVE4 and OVE16 cells (generously donated by Dr. Barbara Vanderhyden, University of Ottawa) were isolated from oviducts of female FVB/N mice [19]. Using these cells, we either deleted the *Trp53* gene (OVE4KO, OVE16KO) through CRISPR/Cas9-mediated genome editing or transduced a gain-of-function p53^R175H^ mutation (OVE4MUT, OVE16MUT). All OVE cells were cultured in Alpha Modification Eagle’s Medium (AMEM; Wisent Inc) with 4% fetal bovine serum (FBS; Thermo Fisher Scientific), 1% antibiotic/antimycotic (ABAM; Wisent Inc), 1 ug/mL insulin-transferrin-sodium-selenite (Sigma-Aldrich), 0.02 ug/mL epidermal growth factor (R&D Systems) and 0.01 nM estradiol (Sigma-Aldrich) and incubated in 5% CO_2_ at 37 °C.

Human FTE cells, FT237 and FT190 (generously donated by Dr. Ronny Drapkin, University of Pennsylvania), were cultured in Dulbecco’s High Glucose Modified Eagle’s Medium and Ham’s F12 mixture (Wisent Inc) with 2% Ultroser G (Pall Corporation) and 1% ABAM (Wisent Inc) and incubated in 5% CO_2_ at 37 °C.

Murine ID8 (generously donated by Drs. Katherine Roby and Paul Terranova, University of Kansas) and 28–2 cells were cultured in Dulbecco’s Modified Eagle Medium (Wisent Inc) with 10% FBS (Thermo Fisher Scientific), 2% L-glutamine (Wisent Inc) and 1% ABAM (Wisent Inc) and incubated in 5% CO_2_ at 37 °C. 28–2 ascites cells were isolated from our ID8 orthotopic, syngeneic murine ovarian cancer model [6]. ID8 cells are p53 wild-type, whereas 28–2 cells have an acquired gain-of-function p53 mutation [6].

### Generation of *Trp53* knockout OVE cell lines

OVE4 and OVE16 cells were seeded in 6-well plates (5.0 × 10^4^ cells/well) until 80% confluent. Cells were transfected with two pSpCas9-Trp53 plasmids (5’-AGTGAAGCCCTCCGAGTGTC-3’ and 5’-AACAGATCGTCCATGCAGTG-3’) using Lipofectamine LTX (Thermo Fisher Scientific) according to the manufacturer’s instructions and incubated overnight. Then, cells were supplemented with complete media with 2 ug/mL puromycin overnight. Transfected *Trp53* knockout OVE cells were expanded in complete media and colonies were grown from single cells. *Trp53* knockout was confirmed by western blotting and clones lacking p53 expression were mixed in equal ratios generating OVE4KO and OVE16KO *Trp53* knockout cell lines.

### Generation of p53^R175H^ mutant OVE cell lines

HEK293T cells were transfected with a p53^R175H^ mutant target vector (pLenti6/V5-p53_R175H, Addgene plasmid) and two viral vectors containing the packaging genes, psPAX2 and pMD2.G, at a ratio of 10:6:4, and incubated for 48 h. The media was collected and filtered using a 0.45um filter (Sigma-Aldrich), then mixed with polybrene (Santa Cruz Biotechnology Inc) and added to parental OVE4 and OVE16 cells and incubated for 24 h. The media was removed, and transfected OVE cells were supplemented with complete media with 2 ug/mL puromycin for 7 days. Transfected OVE cells were expanded in complete media generating OVE4MUT and OVE16MUT p53^R175H^ mutant cell lines.

### mCherry transfection

All OVE cells (5.0 × 10^4^ cells/well) were seeded in 6-well plates. The next day, cells were transfected with a puro-mCherry plasmid (generously gifted by Dr. Sarah K. Wootton, University of Guelph) using polybrene (Santa Cruz Biotechnology Inc) according to the manufacturer’s instructions. Following 24 h, cells were supplemented in complete media for 24 h followed by supplementation of complete media with 2 ug/mL puromycin for 72 h, then mCherry positive OVE cells were expanded.

### Statin preparation

For in vitro experiments, simvastatin (Sigma-Aldrich), atorvastatin (Cayman Chemical), rosuvastatin (Cayman Chemical) and pravastatin (Cayman Chemical) were dissolved in dimethyl sulfoxide (DMSO; Sigma-Aldrich) to 5 mg/mL.

For in vivo experiments, simvastatin (Sigma-Aldrich) was converted to its active form as previously described [20]. Four mg of simvastatin was dissolved in 100uL ethanol, then 150uL 0.1N sodium hydroxide was added and incubated in a 50 °C water bath for 2 h. The total volume was brought to 1 mL with sterile PBS to create the stock solution of 4 mg/mL. The stock solution was diluted in PBS, and the pH was adjusted to 7.0 to create a final concentration of 1 mg/kg. The solution was sterile filtered using a 0.22um filter (Sigma-Aldrich) and aliquots were stored at -20 °C. A PBS control solution was prepared using the same ethanol and sodium hydroxide quantities, without simvastatin.

### Animals

Female FVB/N mice (Charles River Laboratories) were housed at the University of Guelph Central Animal Facility in accordance with the Canadian Council on Animal Care. Following isoflurane anesthesia inhalation, the left ovary was elevated through a dorsal midline incision and mCherry-OVE cells (1.0 × 10^5^ cells in 6uL PBS) were injected into the distal oviduct. To characterize the role of p53 status in ovarian cancer disease progression, mice from each OVE cell line (*n* = 3–4 mice/group, per timepoint) were euthanized at days 15, 30 and 60 post-tumor induction (PTI). Ovarian/tumor tissues were weighed and fixed in 10% neutral buffered formalin, paraffin wax embedded, then transferred onto superfrost plus slides (Thermo Fisher Scientific) and baked at 37 °C overnight.

In a separate cohort, OVE4MUT cells (1.0 × 10^5^ cells in 6uL PBS) were injected into the left distal oviduct and mice (*n* = 8–9 mice/treatment group) received daily intraperitoneal injections of 1 mg/kg simvastatin (Sigma-Aldrich) or PBS at day 25 PTI. At day 50 PTI, mice were euthanized, and ovarian tumors were weighed and either flash frozen in liquid nitrogen and stored at -80 °C or fixed in 10% neutral buffered formalin as previously described.

Mice injected with OVE4MUT cells develop abdominal ascites, characteristic of advanced-stage ovarian cancer in women. A 25-gauge needle was used to aspirate ascites fluid from the peritoneal cavity of these mice. Ammonium-chloride-potassium buffer was used to lyse red blood cells, and after 5 min of centrifugation at 500xG, cells were pelleted. The cells were reconstituted in complete AMEM medium and incubated at 37 °C with 5% CO_2_. When cells reached 70–80% confluency the ascites cells and the original mCherry-OVE4MUT cell line were imaged using an EVOS FL auto imaging system microscope (Life Technologies, California, USA) to confirm the presence of mCherry positive cells.

### Resazurin sodium salt cell viability assay

A resazurin assay was conducted to quantify the impact of statin treatment on cell viability. All OVE cells (3.0 × 10^3^ cells/well; *n* = 3/treatment group) were seeded in 96-well plates. Cells were serum starved for 24 h and then treated with varying doses (0.001uM-1000uM) of simvastatin, atorvastatin, rosuvastatin or pravastatin for 24 h in normal culture medium. Resazurin sodium salt (Sigma-Aldrich) was added at 0.5 mg/mL and incubated for 3 h at 37 °C until data acquisition time point. Metabolic activity was measured using a microplate reader (SpectraMax i3, Molecular Devices; excitation wavelength: 535/9 nm, emission wavelength: 590/15 nm) and the experiment was repeated in triplicate. FT237 (2.5 × 10^3^ cells/well), FT190 (3.0 × 10^3^ cells/well) and ID8 and 28–2 (1.5 × 10^3^ cells/well; *n* = 3/treatment group) were subjected to simvastatin treatment only as previously described. This experiment was performed to determine the most efficacious statin and dose (10 uM simvastatin) for future experiments.

To determine the effect of simvastatin treatment on metabolic activity over time, OVE4 cells (wild-type, p53^R175H^ mutant and *Trp53* knockout) were seeded (3.0 × 10^3^ cells/well *n* = 3/treatment group) in 96-well plates. Cells were serum starved for 24 h and treated with either 10uM simvastatin or DMSO for 3–48 h. Resazurin sodium salt (Sigma-Aldrich) was added, and metabolic activity was measured as previously described.

### Invasion assay

4 × 10^4^ OVE cells (*n* = 3) were seeded in the transwell upper chamber (Corning Inc) in serum free AMEM media in triplicate. The lower chamber contained complete AMEM media with FBS as a chemoattractant and cells were incubated in a humidified 5% CO_2_ atmosphere at 37 °C for 24 h. Following incubation, cells were fixed in methanol for 2 min, stained in 0.5% toluidine blue for 20 min and dried overnight. Four random fields per well were imaged at 40X magnification and the percentage of area invaded was calculated using ImageJ software.

For simvastatin experiments, OVE4 cells (wild-type, p53^R175H^ mutant and *Trp53* knockout; *n* = 3/treatment group) were serum starved for 24 h and then subsequently treated with 10uM simvastatin or DMSO for 24 h. Following treatment, cells were trypsinized and centrifuged to remove treatment media, then resuspended in serum free AMEM media. 4 × 10^4^ cells from each treatment group were seeded in the transwell upper chamber in triplicate and the same protocol was followed as described above.

### Live-cell imaging

OVE cells (3.0 × 10^3^ cells/well; *n* = 3/treatment group) were seeded in 96-well plates. Cells were serum starved for 24 h then treated with 10uM simvastatin or DMSO for 24 h. During the 24-h treatment period, brightfield images were captured every 3 h using an IncuCyte S3 Live-Cell Analysis System (Sartorius) and the experiment was repeated in triplicate. The percentage of cell confluency was then calculated using ImageJ software.

### Caspase-Glo 3/7 and CyQUANT cell proliferation assays

OVE cells (3.0 × 10^3^ cells/well; *n* = 3/treatment group) were seeded in 96-well plates. Cells were serum starved for 24 h and then treated with 10uM simvastatin or DMSO for 24 h. For cell proliferative assays, treatment media was removed and cells were frozen at -80 °C then thawed to lyse cells. CyQUANT dye reagent was prepared and added to the wells as per the manufacturer’s instructions (Thermo Fisher Scientific). Cells were frozen at -80 °C again, then thawed and incubated for 10 min at room temperature. Proliferative activity was measured using a microplate spectrophotometer (Wallac 1420 Victor 2; PerkinElmer; excitation wavelength: 480/9 nm, emission wavelength: 520/15 nm) and the experiment was repeated in triplicate.

For apoptotic assays, Caspase-Glo 3/7 reagent (Promega) was prepared and added to the wells as per the manufacturer’s instructions and incubated for 30 min on a plate rocker, then 30 min at room temperature. Apoptotic activity was measured by luminescence using a plate reader (BioTek) and the experiment was repeated in triplicate with Caspase-Glo 3/7 luminescence normalized to CyQUANT fluorescence.

### Western blot analysis

OVE cells (*n* = 4/cell line) were lysed in radioimmunoprecipitation assay buffer with protease and phosphatase inhibitors. For simvastatin experiments, 50 mg of PBS and simvastatin-treated flash frozen tumors (*n* = 3/treatment group) were subjected to nuclear and cytoplasmic fractionation as per the manufacturer’s instructions (BioVision Inc.). The protein concentration was then determined by DC Bio-Rad protein assay (Bio-Rad).

Twenty ug of protein was denatured and subjected to SDS-PAGE using 4–15% stain-free gels (Bio-Rad), and then transferred to nitrocellulose membranes (Bio-Rad, Ontario, Canada) with a Trans-Blot Turbo System (Bio-Rad). Blots were blocked for 1 h at room temperature in 5% skim milk or BSA in tris-buffered saline plus 0.1% tween 20. Membranes were incubated overnight at 4 °C with the primary antibodies: anti-SREBP-2 (1:500, Abcam), anti-HMGCR (1:1000, Abcam), anti-Rho (1:500, Abcam), anti-YAP (1:1000, Abcam), anti-p53 (1:1000; R&D Systems, Minnesota, USA), and anti-TAZ (1:500, Abcam). Then, membranes were incubated with the species-appropriate IgG horseradish peroxidase-linked secondary antibody (1:2000, Cell Signaling Technology) for 1 h at room temperature. Expression of proteins of interest was visualized using Clarity Western ECL Blotting Substrate (Bio-Rad) on the ChemiDoc MP System (Bio-Rad). Densitometric analysis was performed using Image Lab software (Bio-Rad) and protein expression for antibodies of interest were normalized to stain-free protein.

### Immunohistochemistry/immunofluorescence

Paraffin-embedded murine tissues (*n* = 6/treatment group) were deparaffinized in xylene and rehydrated in decreasing ethanol concentrations. To analyze tissue morphology, tissues were stained with hematoxylin and eosin (H&E), then mounted on coverslips (Thermo Fisher Scientific) with Cytoseal-XYL (Thermo Fisher Scientific).

For immunohistochemical analysis, endogenous peroxidase activity was blocked by 10-min incubation with 1% hydrogen peroxide followed by antigen retrieval using citrate buffer at 90 °C for 12 min. Tissues were blocked in 5% BSA for 10 min at room temperature, then incubated with anti-mCherry (1:500, Abcam), anti-Rho (1:100, Abcam) and anti-Rac1 (1:100, Novus Biologicals) primary antibodies overnight at 4 °C. Next, slides were incubated with the species-appropriate biotinylated secondary antibody (1:100, Sigma-Aldrich) for 2 h at room temperature, then incubated with extravidin-peroxidase (1:50, Sigma-Aldrich) PBS for one hour at room temperature. To visualize antigens, tissues were incubated with 3,3’-diaminobenzidine (DAB; Sigma-Aldrich) for up to 5 min, counterstained with Carazzi’s hematoxylin then mounted on coverslips (Thermo Fisher Scientific) with Cytoseal-XYL (Thermo Fisher Scientific). All slides were imaged using a brightfield Nikon Eclipse E600 microscope and QCapture software. Four random fields of view were used for image analysis for each tissue. Aperio ImageScope 12.1 software (Leica Biosystems) was used for quantification of cytoplasmic staining.

For immunofluorescence labeling, tissues were blocked in 5% BSA in PBS for 10 min at room temperature and then incubated with anti-mCherry (1:500, Abcam) primary antibody overnight at 4 °C. Next, slides were incubated with the species-appropriate Alexa-fluor fluorescently tagged secondary antibody (1:100, Thermo Fisher Scientific, Massachusetts, USA) for 2 h at room temperature, then mounted on coverslips (Thermo Fisher Scientific, Massachusetts, USA) and nuclei were stained using a ProLong Gold antifade mountant with DAPI (Thermo Fisher Scientific, Massachusetts, USA). All slides were imaged using an Olympus inverted epifluorescence microscope and Metamorph integrated morphometry software (Molecular Devices, California, USA).

In vitro, OVE, FT237, FT190, ID8 and 28–2 cells (2.0 × 10^4^ cells per well) were seeded on 24 mm glass cover slips (VWR) until 80% confluent. Cells were fixed in 10% neutral buffered formalin for 1 h at room temperature, then permeabilized with 0.2% (v/v) Triton X 100 (Sigma-Aldrich) for 5 min. Cells were blocked with 5% BSA for 1 h at room temperature, then incubated overnight at 4 °C with anti-PAX2 (1:500, Abcam) and anti-PAX8 (1:500, Novus Biologicals). Next, cells were incubated with the species-appropriate Alexa-fluor fluorescently tagged secondary antibody (1:1000, Thermo Fisher Scientific) for 1 h at room temperature, then mounted onto slides (Thermo Fisher Scientific) and nuclei were stained using a ProLong Gold antifade mountant with DAPI (Thermo Fisher Scientific). All slides were imaged using an Olympus inverted epifluorescence microscope and Metamorph integrated morphometry software (Molecular Devices).

### Terminal deoxynucleotidyl transferase dUTP nick end labeling (TUNEL)

Paraffin-embedded murine tissues (*n* = 6/treatment group) were deparaffinized in xylene and rehydrated in decreasing ethanol concentrations. Tissues were incubated with TUNEL reaction reagent (Sigma-Aldrich) for 1 h at 37 °C. Tissues were then mounted on coverslips (Thermo Fisher Scientific) and nuclei were stained using a ProLong Gold antifade mountant with DAPI (Thermo Fisher Scientific). All slides were imaged using an Olympus inverted epifluorescence microscope and Metamorph integrated morphometry software (Molecular Devices). Four random fields of view were used for image analysis for each tissue. ImageJ software was used for quantification of fluorescently stained nuclei.

### Statistical analysis

GraphPad Prism v7 (GraphPad software) was used for statistical analysis. Data from untreated in vitro experiments was analyzed using a one-way analysis of variance (ANOVA) and a Tukey’s post hoc test was used to determine statistical differences between groups. For in vitro statin experiments and in vivo murine disease characterization experiments, a two-way ANOVA and a Tukey’s post hoc test was used to determine statistical differences between groups. In vivo murine statin experiments were statistically analyzed using an unpaired t test, apart from western blotting experiments statistically analyzed using a one-way ANOVA and a Tukey’s post hoc test. All reported *p*-values were considered significant at *p* ≤ 0.05, with graphs representing means ± standard error of the mean (SEM).

## Results

### Loss of wild-type p53 does not influence OVE cell invasion, but contributes to increased mevalonate pathway signaling and downstream effects

**Fig. 1:**
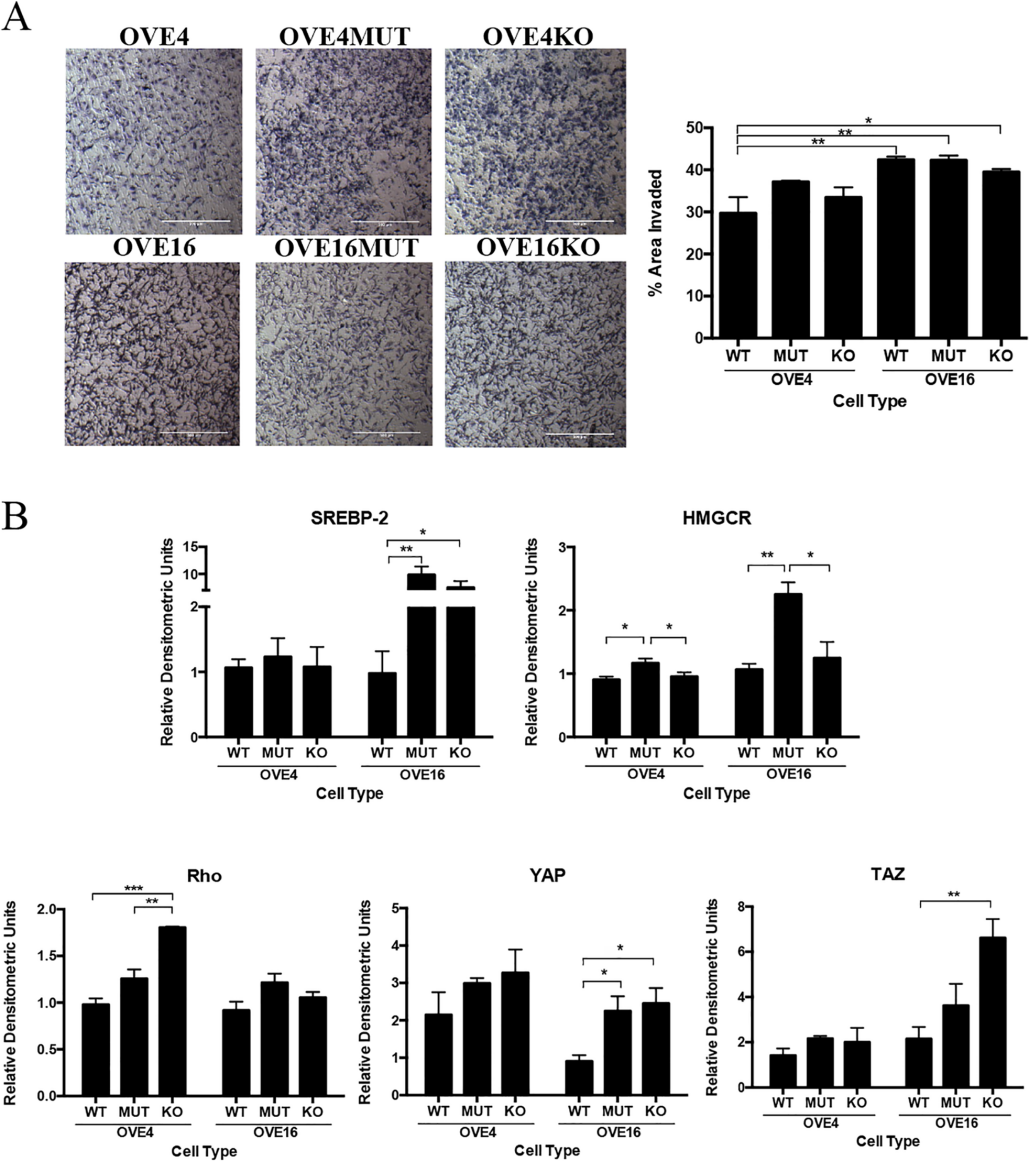
p53 status does not influence cell migration or invasion, but does influence mevalonate pathway expression in OVE cells. **A** OVE cells were seeded and incubated for 24 h in transwell chambers (*n* = 3/group) then stained with toluidine blue and the percent area invaded was calculated. **B** Protein was collected from OVE cells (*n* = 4/group) and western blotting was performed for mevalonate pathway members. Bars represent mean ± SEM (*p < 0.05, **p < 0.01, ***p < 0.001). Scale bars: 500 μm. Abbreviations: WT wild-type; MUT p53^R175H^ mutant; KO *Trp53* knockout

First, we confirmed the p53 status and fallopian tube markers of all cell lines (Supplementary Fig. 1), and then evaluated the role of p53 status on OVE cell migration and invasion capacity. There were no significant differences within each of the OVE4 and OVE16 cell lines (wild-type, p53^R175H^ mutant and *Trp53* knockout), however, OVE16 (*p* < 0.01), OVE16MUT (*p* < 0.01) and OVE16KO (*p* < 0.05) cells had greater invasion compared to OVE4 p53 wild-type cells (Fig. 1A), demonstrating changing the p53 status of OVE cells is not sufficient to increase migration and invasion properties.

We next evaluated p53 status influence on mevalonate pathway signaling in OVE cells. Sterol regulatory element binding protein 2 (SREBP-2) is responsible for transcribing many genes of the mevalonate pathway, including the pathway’s rate-limiting enzyme, HMGCR. Wild-type p53 blocks activation of SREBP-2 to decrease mevalonate pathway activity, however in cancer, mutant p53 and SREBP-2 upregulate transcription of key mevalonate genes. We demonstrated that SREBP-2 expression was similar between OVE4 cell lines (wild-type, p53^R175H^ mutant, *Trp53* knockout), however, OVE16MUT (*p* < 0.01) and OVE16KO (*p* < 0.05) cells had elevated SREBP-2 expression compared to p53 wild-type OVE16 cells (Fig. 1B). Additionally, OVE4MUT and OVE16MUT cells had increased HMGCR expression compared to their respective wild-type and *Trp53* knockout cell lines (*p* < 0.05; Fig. 1B).

The mevalonate pathway also regulates small GTPase protein prenylation, including increased Rho expression in numerous cancers [21]. Here, OVE4KO cells had was significantly increased Rho expression compared to OVE4MUT (*p* < 0.01) and wild-type OVE4 (*p* < 0.001) cells, with no significant changes in Rho expression between OVE16 cells (wild-type, p53^R175H^ mutant, *Trp53* knockout; Fig. 1B). Downstream of the mevalonate pathway, YAP/TAZ from the Hippo pathway are activated in many cancers [22]. Both p53 wild-type OVE4 and OVE16 cells had the lowest expression of YAP/TAZ, with significant increases in YAP (*p* < 0.05) and TAZ expression in OVE16KO (*p* < 0.01) cells (Fig. 1B). Overall, p53 wild-type OVE cells demonstrated the lowest expression for each antibody of interest (Fig. 1B).

### p53^R175H^ mutant OVE cells induce ovarian cancer progression in vivo

**Fig. 2:**
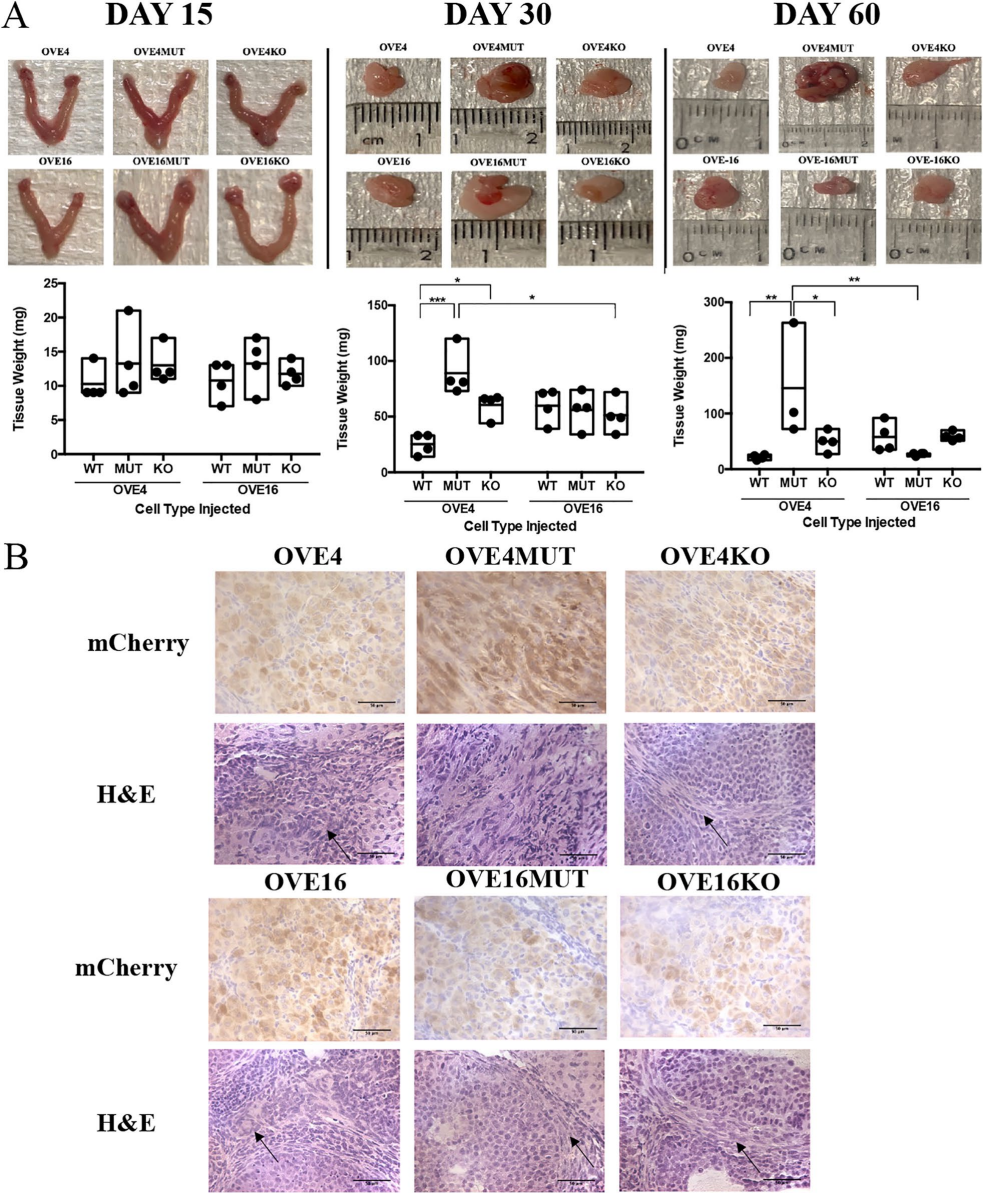
OVE cells colonize on the ovarian surface and p53^R175H^ mutant OVE cells form ovarian tumors and progress to advanced-stage disease. **A** mCherry-OVE cells were injected into the left oviduct of female FVB/N mice and ovarian/tumor tissues were collected and weighed at 15, 30 and 60 days PTI (*n* = 3–4/group). **B** Immunohistochemical mCherry and H&E staining were performed on tumors collected at day 60. Arrows point to serous tissue (*p < 0.05, **p < 0.01, ***p < 0.001). Scale bars: 50 μm. Abbreviations: WT wild-type; MUT p53^R175H^ mutant; KO *Trp53* knockout

We evaluated p53 status of OVE cells on ovarian cancer disease progression in vivo. All mCherry-OVE (wild-type, p53^R175H^ mutant and *Trp53* knockout) cells were injected into the left oviduct of female FVB/N mice. At day 15, there were no observable differences in disease progression (Fig. 2A). However, at day 30, mice injected with OVE4MUT p53^R175H^ mutant cells had significantly larger tumors compared to wild-type OVE4 tumors (*p* < 0.001) and OVE16KO tumors (*p* < 0.05; Fig. 2A). Additionally, OVE4KO tumors were significantly larger than wild-type OVE4 tumors (*p* < 0.05; Fig. 2A). By day 60, mice injected with OVE4MUT cells had significantly larger tumors compared to wild-type OVE4 (*p* < 0.01), OVE4KO (*p* < 0.05) and OVE16MUT tumors (*p* < 0.01; Fig. 2A).

Tissues collected on day 60 were subjected to H&E and immunohistochemical staining for mCherry. mCherry positive staining confirmed the presence of OVE cells on the ovarian surface (Fig. 2B), characteristic of the fallopian tube origin of HGSC. H&E staining demonstrated formation of serous tissue by all OVE cells, however, OVE4MUT tumors were primarily all serous tumor tissue (Fig. 2B). Additionally, OVE4MUT cells progressed to advanced-stage disease, yet OVE16MUT cells did not progress in the same way (Fig. 2). As such, we further characterized our disease model of HGSC using OVE4MUT cells for tumor induction (Supplementary Fig. 2).

### Simvastatin more potently inhibits metabolism than other statins in OVE cells, regardless of p53 status

**Fig. 3:**
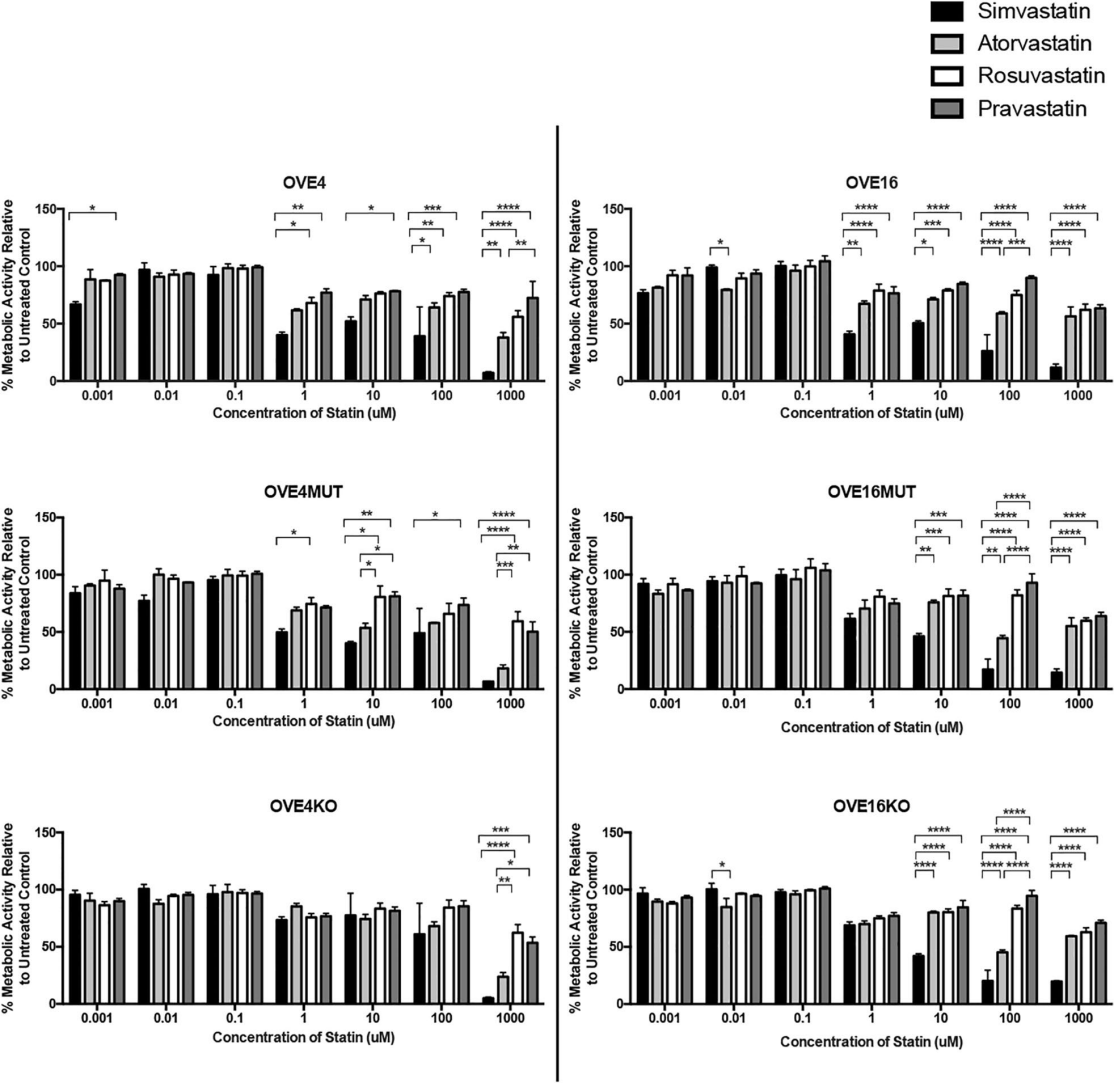
OVE cells are most sensitive to metabolic inhibition with simvastatin treatment. OVE cells (*n* = 3/group) were treated with varying doses (0.001uM-1000uM) of simvastatin, atorvastatin, rosuvastatin or pravastatin for 24 h and then subjected to a resazurin sodium salt cell metabolic assay. Bars represent mean ± SEM (*p < 0.05, **p < 0.01, ***p < 0.001, ****p < 0.0001)

We investigated the effect of lipophilic (simvastatin and atorvastatin) and hydrophilic (rosuvastatin and pravastatin) statins on OVE cell metabolic activity. Both lipophilic statins were more efficacious than hydrophilic statins in all OVE cells, regardless of p53 status (Fig. 3). Simvastatin markedly reduced metabolic activity at a dose as low as 1uM compared to other statins (*p* < 0.05; Fig. 3). At higher concentrations, simvastatin was most efficacious, followed by atorvastatin, rosuvastatin and lastly pravastatin (Fig. 3). Based on these results, and previous research by our lab (6), simvastatin was selected for future experiments.

### Simvastatin demonstrates a dose-dependent inhibitory response in vitro

**Fig. 4:**
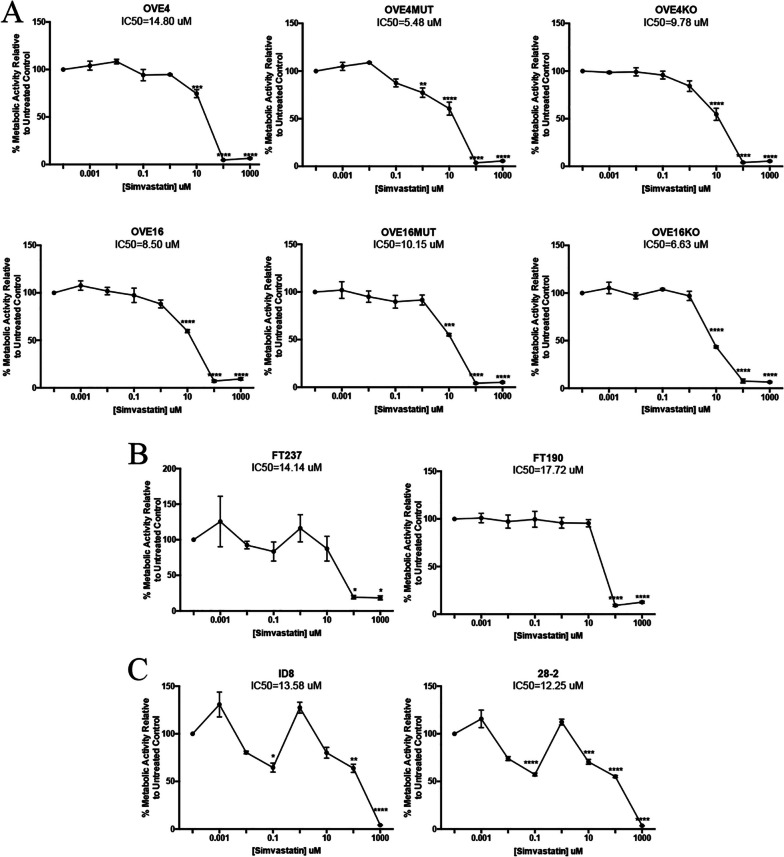
p53^R175H^ mutant OVE cells are more susceptible to simvastatin treatment compared to wild-type OVE cells and ovarian surface epithelial cells. **A** OVE (wild-type, p53^R175H^ mutant and *Trp53* gene knockout), **B** human FTE cells and **C** ovarian surface epithelial cells (*n* = 3/group) were treated with varying doses (0.00uM-1000uM) of simvastatin for 24 h and subjected to a resazurin sodium salt cell metabolic assay, then IC50 values were calculated. Bars represent mean ± SEM (*p < 0.05, **p < 0.01, ***p < 0.001, ****p < 0.0001)

Following a metabolic resazurin cell assay, all cell lines demonstrated sensitivity to simvastatin in a dose-dependent manner (Fig. 4). The half-maximal inhibitory concentration (IC50) was calculated and OVE4MUT cells had the lowest IC50 value and reduced metabolic rate at a dose as low a 1uM (*p* < 0.01; Fig. 4A). OVE16MUT cells showed less sensitivity to simvastatin through a higher IC50 value and metabolic changes at a dose as low as 10uM (*p* < 0.001; Fig. 4A). Human FTE cells had similar IC50 values with FT237 cells having a lower IC50 value compared to FT190 cells (Fig. 4B). Ovarian surface epithelial cells were less sensitive to simvastatin with higher IC50 values than most OVE cells (Fig. 4C).

### Simvastatin inhibits proliferation, invasion, migration and metabolic activity and promotes apoptosis of OVE cells

**Fig. 5:**
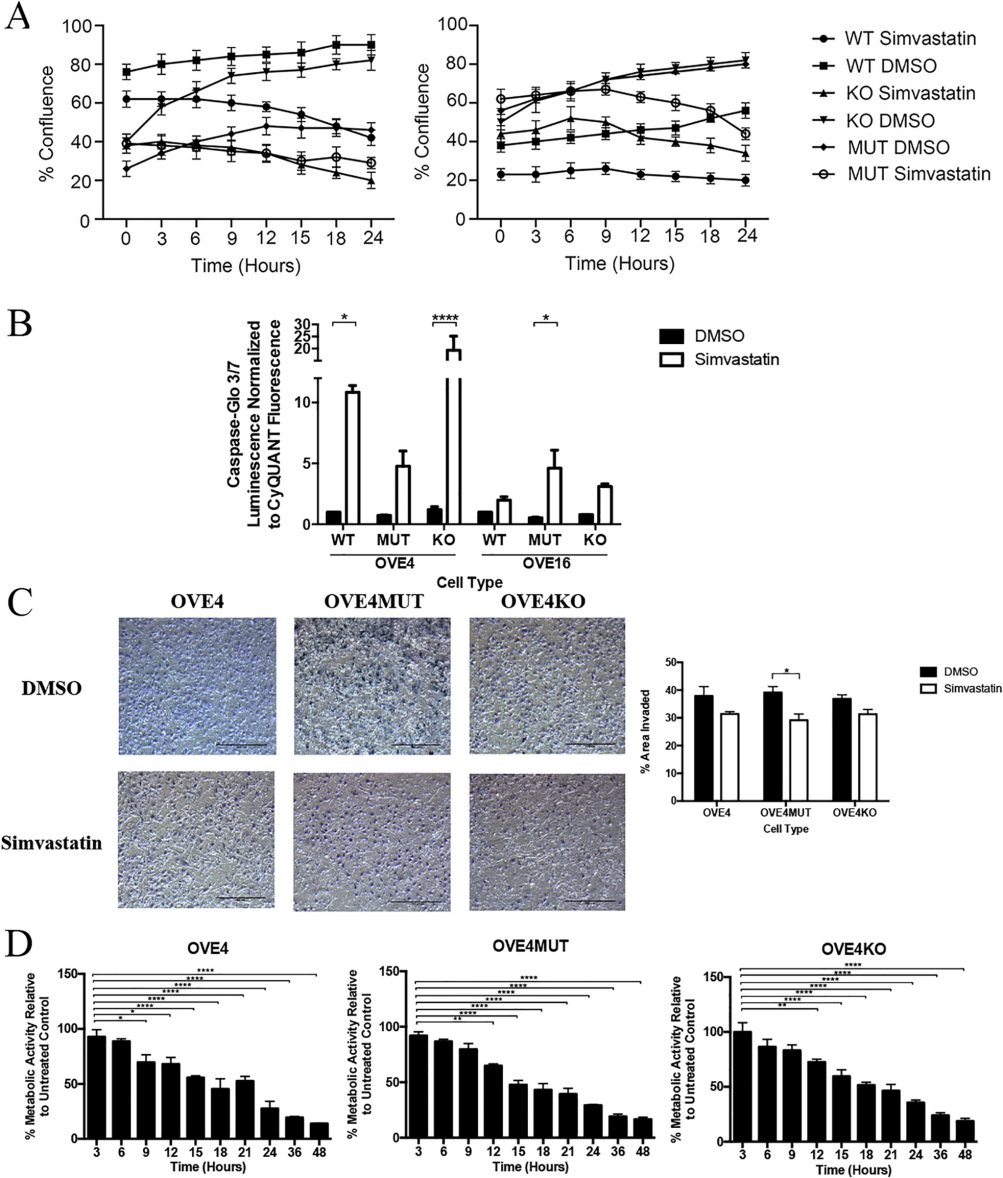
Simvastatin influences cell proliferation, migration, invasion, apoptosis and metabolism in OVE cells. OVE cells (*n* = 3/group) were treated with 10uM simvastatin or DMSO for all experiments. **A** OVE cells were treated for 24 h, brightfield images were captured using an IncuCyte S3 Live-Cell Analysis System and the percent confluency at each timepoint was calculated. **B** OVE cells were treated for 24 h then subjected to the Caspase-Glo 3/7 apoptotic assay and normalized to CyQUANT proliferative assay values. **C** OVE4 (wild-type, p53^R175H^ mutant and *Trp53* knockout) cells were treated for 24 h, then seeded and incubated for 24 h in transwell chambers. Cells were stained with toluidine blue and the percent area invaded was calculated. **D** OVE4 (wild-type, p53^R175H^ mutant and *Trp53* knockout) cells were treated for 3–48 h and subjected to a resazurin sodium salt cell metabolic assay. Bars represent mean ± SEM, (*p < 0.05, **p < 0.01, ***p < 0.001, ****p < 0.0001). Scale bars: 500 μm. Abbreviations: WT wild-type; MUT p53^R175H^ mutant; KO *Trp53* knockout

We investigated simvastatin’s effect on tumorigenic properties in vitro. As rapid proliferation is characteristic of cancer cells, we used an IncuCyte to investigate OVE cell proliferation following simvastatin treatment. Over 24-h, simvastatin-treated cells had slower proliferation based on the percent confluency of cells (Fig. 5A). Next, we evaluated simvastatin’s effect on apoptosis through caspase-3/7 activity. All simvastatin treated OVE cells had increased caspase-3/7 activity, however this was only significant for OVE4 (*p* < 0.05), OVE4KO (*p* < 0.0001) and OVE16MUT cells (*p* < 0.05; Fig. 5B).

As OVE4MUT cells were the only cell line to progress to advanced-stage disease in vivo, we focused future experiments using OVE4 (wild-type, p53^R175H^ mutant or *Trp53* knockout) cells. We assessed simvastatin’s influence on cell invasion and migration, and simvastatin decreased OVE cell Matrigel invasion, with decreased capacity in OVE4MUT cells (*p* < 0.05; Fig. 5C). As simvastatin targets the mevalonate pathway, a metabolic pathway, we evaluated OVE cell metabolic activity as measured by aerobic respiration of resazurin reduction to resorufin over a 48-h period of simvastatin treatment. We demonstrated significantly decreased metabolic activity in all OVE cells as early as 9 h of simvastatin treatment (*p* < 0.05; Fig. 5D).

### Simvastatin reduces tumor size and increases apoptosis in vivo

**Fig. 6:**
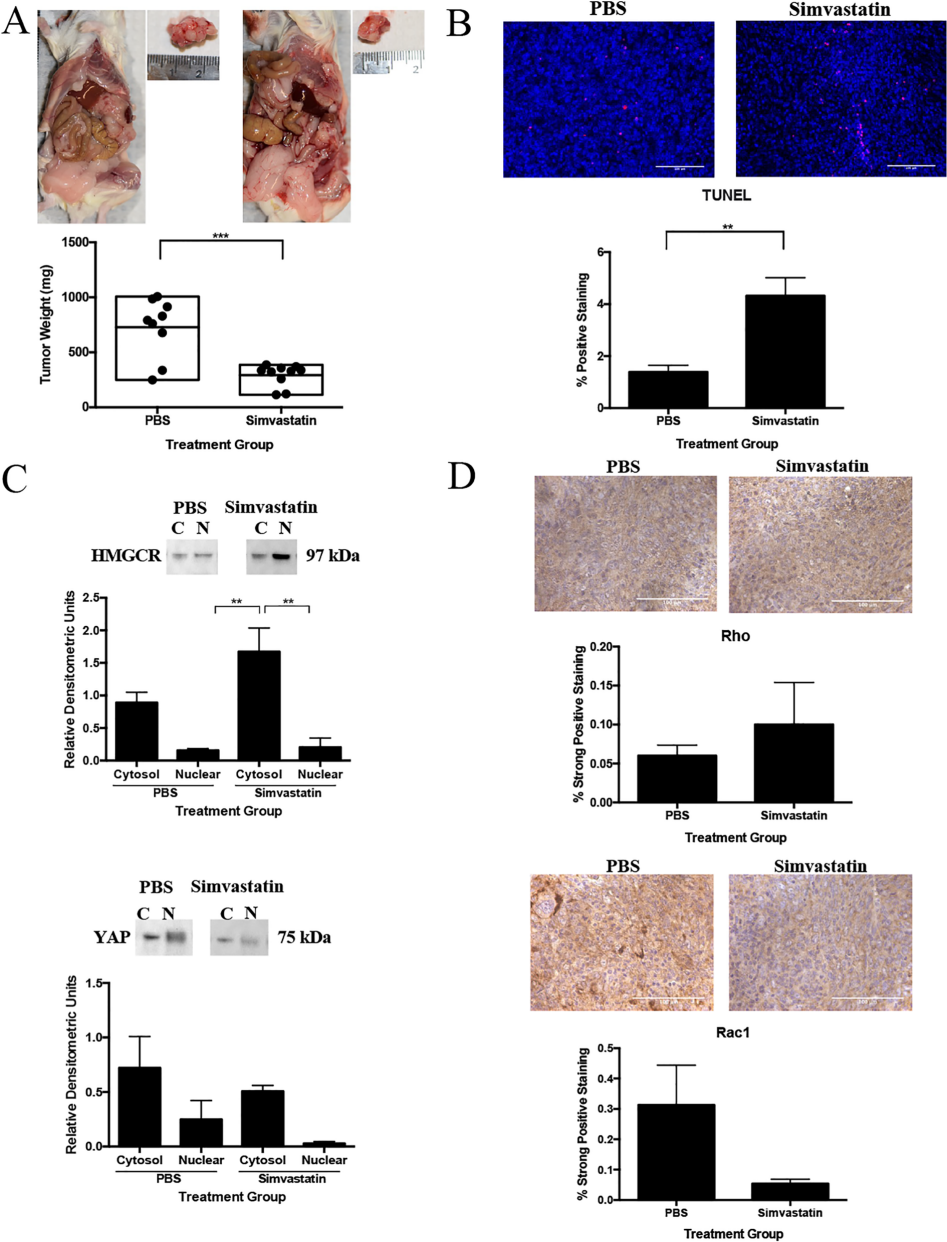
Simvastatin inhibits ovarian cancer disease progression, increases apoptosis and HMGCR cytoplasmic localization, but does not influence YAP or small GTPase expression. **A** Mice were subjected to daily intraperitoneal injections of simvastatin (1 mg/kg) or PBS for 25 days, then tumors were collected and weighed (*n* = 8–9/group). **B** TUNEL staining (*n* = 6/group) was performed on PBS and simvastatin treated tumors. **C** Cytoplasmic and nuclear protein fractions were collected from flash-frozen PBS and simvastatin treated tumors (*n* = 3/group) and western blotting was performed for HMGCR and YAP. **D** PBS and simvastatin treated tumors (*n* = 6/group) were immunohistochemically stained for the small GTPases, Rho and Rac1. Bars represent mean ± SEM (**p < 0.01, ***p < 0.001). Scale bars: 100 μm. Abbreviations: C cytoplasmic; N nuclear

Using our OVE4MUT cells, we evaluated simvastatin’s effect on disease regression. Simvastatin treated mice had significantly smaller ovarian tumors, compared to PBS mice (*p* < 0.001, Fig. 6A). We then investigated simvastatin’s effect on single- and double-stranded DNA fragmentation via TUNEL, indicative of apoptosis. Simvastatin treated tumors had a significant increase (*p* < 0.01) in TUNEL positive staining compared to PBS tumors (Fig. 6B).

### Simvastatin localizes HMGCR to the cytoplasm, but does not significantly influence YAP or GTPase activity

Simvastatin treated tumors had significant cytoplasmic accumulation of HMGCR compared to nuclear fractions from PBS and simvastatin tumors (*p* < 0.01; Fig. 6C). Simvastatin treated tumors had little nuclear and decreased cytoplasmic YAP expression compared to PBS fractions, however, this was not significant (Fig. 6C). We then investigated the relationship between statins and small GTPases. Simvastatin treated tumors demonstrated increased Rho and decreased Rac1 expression, however, this was not significant (Fig. 6D).

## Discussion

We developed an orthotopic, syngeneic FTE-induced HGSC mouse model that replicates disease progression characteristics in women. We observed that p53^R175H^ mutant OVE cells accelerated disease progression. In concert with enhanced mevalonate activity, these cells were particularly susceptible to pathway inhibition with simvastatin.

Gain of function p53^R175H^ mutation binds and activates SREBP-2, increasing its transcriptional activity of mevalonate enzymes, including HMGCR, acting as a constant “on switch” [23, 24], which drives cancer cell proliferation and inhibits apoptosis [6]. As such, the development of a p53 mutation appears to be an initiating event in upregulating the mevalonate pathway as a survival mechanism. Inhibition of the mevalonate pathway by statin drugs can reverse this p53 mutation-induced pathway activation to inhibit the protumorigenic influence [6]. In our OVE cell lines, we saw some discrepant results related to p53 status. In addition to p53, there are other regulators of SREBP-2. mTORC1 is a regulator of lipid metabolism and suppresses autophagy and, through endosomal recycling, prevents membrane-derived cholesterol from reaching lysosomes, reducing cholesterol levels and activating SREBP-2 [25]. In breast cancer cells, mTORC1 activates SREBP-2 to promote lipogenesis, cell growth, proliferation and survival that can be ablated with mTOR inhibitors, linking mTORC1 control to SREBP-2 signaling [26]. This correlates mTORC1 expression and upregulated SREBP-2 activity in cancer, providing a substitute for mutant p53-mediated SREBP-2 regulation. Additionally, the AMP kinase-related protein, salt-inducible kinase 2, upregulates SREBP-1 and SREBP-2 in ovarian cancer cells, through increased fatty acid and cholesterol synthesis, demonstrating a regulatory role of SREBP-2 through PI3K/Akt signaling [27]. These various regulators of SREBP-2 may explain the discrepancy between expression in our OVE p53^R175H^ mutant cell lines.

Regulation of post-translational modifications of small GTPases and YAP/TAZ nuclear localization are important in cancer progression [28, 29]. In our OVE4 cells, we observed a significant increase in Rho expression in the p53 mutant and knockout cells, despite no increase in SREBP-2, suggesting that Rho activation may be influenced by a SREBP-2-independent mechanism. In addition to p53-mevalonate signaling, Myc and microRNAs (miRNAs) also regulate GTPases and YAP/TAZ activity. In head and neck squamous cell carcinoma, VAV2 activity forms a Rho-Myc-YAP signaling axis responsible for promoting a stem cell-like regenerative proliferation of tumor cells [30]. Additionally, the tumor suppressor miRNAs, miR-9-3p, -375 and -582-5p, enhance YAP/TAZ inactivation through phosphorylation and cytoplasmic retention [31–33]. YAP/TAZ are also implicated in sustaining a positive feedback loop, predominantly through TAZ-mediated overexpression of several miRNAs, including miR-135b-5p and -942-3p, to promote epithelial-mesenchymal transition, proliferation, angiogenesis and homeostasis of glycometabolism and reactive oxygen species [34, 35]. In light of this, wild-type p53 maintains tight control on the mevalonate pathway and downstream signaling, and this regulation is ablated by loss of p53 function.

The immune system is imperative to the ovarian microenvironment and mutant p53 thrives in an inflammatory environment. Inflammation is linked to cancer associated fibroblasts and tumor-associated macrophages to drive epithelial-mesenchymal transition and metastasis [36]. STIC lesions progress to HGSC through acquisition of pro-tumorigenic properties including increased proliferation, nuclear polymorphisms and loss of epithelial polarity [37]. Given this, p53 status alone may not solely initiate HGSC, and co-occurring mutations in *BRCA, PTEN,* or other genes may enhance disease initiation [38, 39]. Our two p53^R175H^ mutant OVE cells harbour a gain-of-function *Trp53* mutation, without other perturbations. In light of this, a plausible cause for differences in disease development between our two p53^R175H^ mutant OVE cells may be facilitated by acquired mutations from the ovarian microenvironment.

The structural differences between lipophilic and hydrophilic statins may explain the differences in OVE cell sensitivity. Lipophilic statins passively diffuse through all cell membranes, whereas hydrophilic statins require active transport and are localized to the liver [40], therefore lipophilic statins spread effortlessly through a variety of tissues, including tumor cells. This compliments observational studies on statin use following ovarian cancer diagnosis, where lipophilic statin use was associated with reduced mortality in patients [18], supporting our choice to evaluate simvastatin’s effect on HGSC.

Tumor TUNEL fluorescence demonstrated an increase in simvastatin-induced DNA strand breaks, indicative of apoptosis. Other researchers demonstrated human ovarian cancer cells treated with simvastatin had increased caspase-3/7 activity and decreased pro-survival Bcl-2 and survivin mRNA levels [10]. Simvastatin-treated human SKOV3 cells also experience G1 phase cell cycle arrest and enhanced mitochondrial cytochrome c release to promote apoptosis [41]. Outside of ovarian cancer, simvastatin inhibits heat shock protein 90 and enhances caspase-3/8/9 activity in a murine breast cancer xenograft model [42]. Based on our findings both in vitro and in vivo and other research, simvastatin may induce tumor cell apoptosis independent of caspase-3 activity.

Previous reports have confirmed statin treatment induced accumulation of HMGCR instead of its degradation, coupled with decreased transcription and mRNA levels of HMGCR [43], with YAP expression being significantly downregulated in human pancreatic cancer cells following statin treatment [44]. Additionally, approximately 75% of ovarian cancer patients respond to initial chemotherapies, however most experience disease recurrence and chemoresistance [45]. YAP activation is associated with chemoresistance in ovarian and other cancers [46–48]. As such, the use of simvastatin in combination with chemotherapy may strategically target YAP signaling to prevent initial chemoresistance. Similarly, other researchers have demonstrated statins do not necessarily degrade GTPases, but rather influence their localization primarily by membrane to cytoplasmic shuttling [49]. Most Rho GTPases within the cytosol are bound to Rho guanine nucleotide dissociation inhibitors (RhoGDIs), regulating Rho stability to prevent degradation [50]. Based on this study and other supporting literature, simvastatin appears to alter switching between nuclear and cytoplasmic localization of these proteins rather than eliminating their expression.

Simvastatin substantially decreased tumor burden and increased tumor cell apoptosis in a pre-clinical FTE-driven model of HGSC. Therefore, simvastatin necessitates further research to characterize its mechanism and evaluate if simvastatin has an added anti-tumor benefit when used in a combinational multimodal approach.

### Supplementary Information

**Additional file 1: Supplemental Figure 1.** Confirmation of p53, PAX2 and PAX8 expression in murine oviductal epithelial (OVE) cells, human fallopian tube epithelial cells, spontaneously transformed murine ovarian surface epithelial cells and metastatic ascites cells. A. Protein was collected from OVE and human fallopian tube epithelial cells (*n* = 4/group) and western blotting was performed to confirm p53 expression in p53^R175H^ mutant OVE cells (OVE4MUT, OVE16MUT. Wild-type p53 cells (OVE4, OVE16, FT237) and *Trp53* knockout OVE cells (OVE4KO, OVE16KO) have no p53 expression, confirming normal wild-type function or *Trp53* gene knockout. The transcription factors PAX2 and PAX8 are characteristic to fallopian tube development, but are not expressed in the ovary. Immunofluorescence staining confirms (B) PAX2 and (C) PAX8 staining in all murine OVE and human fallopian tube epithelial cells. Additionally, murine ID8 and 28-2 cells, which are of ovarian surface epithelial origin, have minimal to no expression of PAX2 and PAX8. Bars represent mean ± SEM (*p < 0.05, ****p < 0.0001). Scale bars: 50μm. Abbreviations: WT wild-type; MUT p53 mutant; KO *Trp53 *knockout.

**Additional file 2: Supplemental Figure 2.** Development of an immunocompetent, orthotopic, syngeneic murine model replicating oviductal origin of HGSC. A. mCherry-OVE4MUT cells were injected into the left oviduct of female FVB/N mice and tumor tissues were collected and weighed at 15, 30 and 60 days PTI (*n* = 3–4/group). Immunofluorescence staining for mCherry and H&E staining was performed on murine ovarian tumors collected at all timepoints. B. Confirmation of mCherry staining in OVE4MUT cells and aspirated ascites cells and immunofluorescence staining for mCherry on murine ovarian and secondary metastatic tumors. Images of cells were taken at 200X and images on tissues were taken at 400X. Arrows point to serous tissue in H&E staining. Bars represent mean ± SEM (*p < 0.05). Scale bars: 100 μm (H&E), 50μm (immunofluorescence-tumor); 200μm (cells).

**Additional file 3.**

## Data Availability

All data are included in the manuscript.
