# Supplementary figures and images for: Mutant p53 murine oviductal epithelial cells induce progression of high-grade serous carcinoma and are most sensitive to simvastatin therapy in vitro and in vivo
Source: J Ovarian Res. 2023 Nov 20;16:218. doi: 10.1186/s13048-023-01307-x (PMC10662458; doi:10.1186/s13048-023-01307-x)

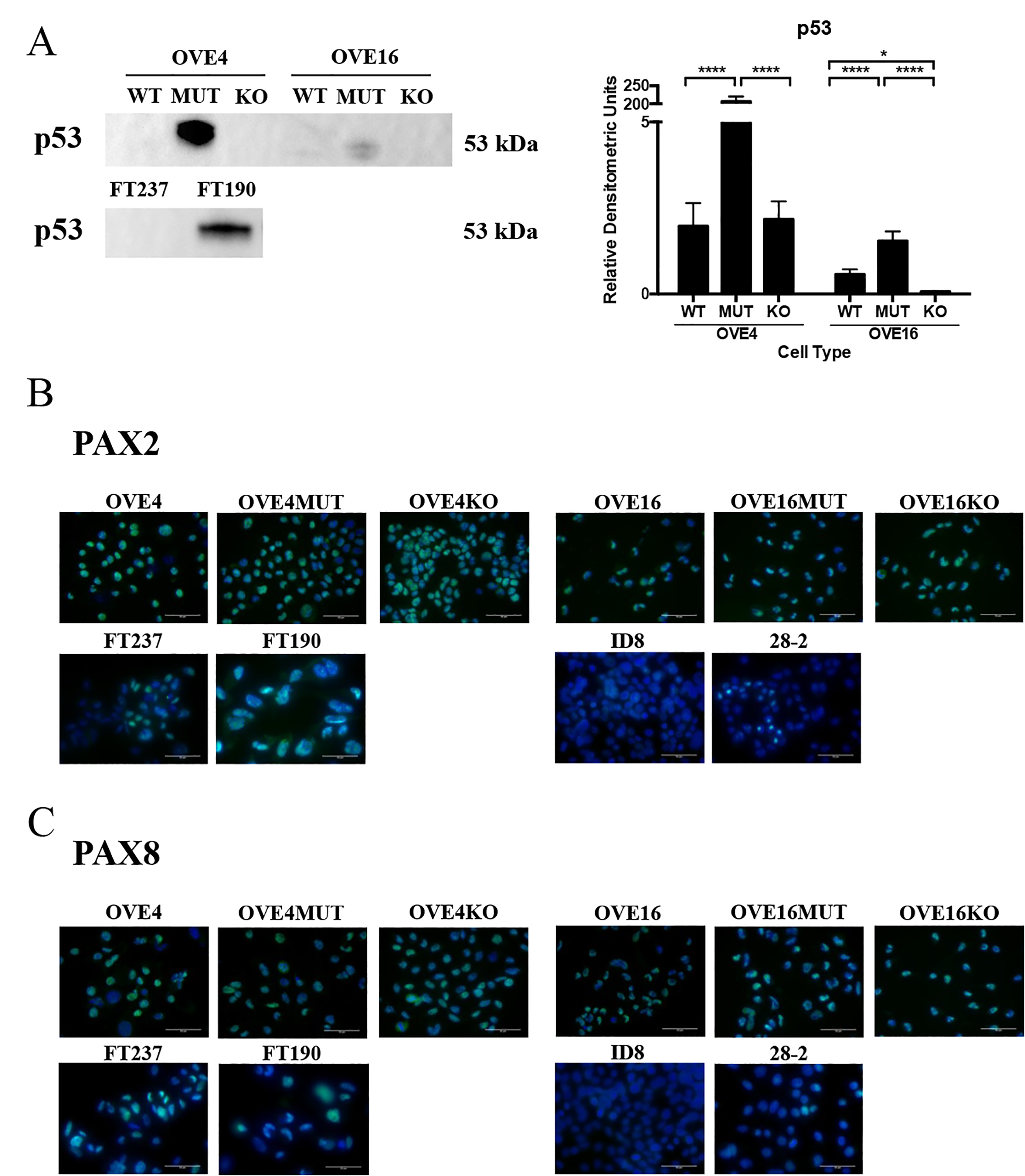

Supplement: Supplementary file 1 — Additional file 1: Supplemental Figure 1. Confirmation of p53, PAX2 and PAX8 expression in murine oviductal epithelial (OVE) cells, human fallopian tube epithelial cells, spontaneously transformed murine ovarian surface epithelial cells and metastatic ascites cells. A. Protein was collected from OVE and human fallopian tube epithelial cells (n = 4/group) and western blotting was performed to confirm p53 expression in p53R175H mutant OVE cells (OVE4MUT, OVE16MUT. Wild-type p53 cells (OVE4, OVE16, FT237) and Trp53 knockout OVE cells (OVE4KO, OVE16KO) have no p53 expression, confirming normal wild-type function or Trp53 gene knockout. The transcription factors PAX2 and PAX8 are characteristic to fallopian tube development, but are not expressed in the ovary. Immunofluorescence staining confirms (B) PAX2 and (C) PAX8 staining in all murine OVE and human fallopian tube epithelial cells. Additionally, murine ID8 and 28-2 cells, which are of ovarian surface epithelial origin, have minimal to no expression of PAX2 and PAX8. Bars represent mean ± SEM (*p < 0.05, ****p < 0.0001). Scale bars: 50μm. Abbreviations: WT wild-type; MUT p53 mutant; KO Trp53 knockout. [file 13048_2023_1307_MOESM1_ESM.tif]

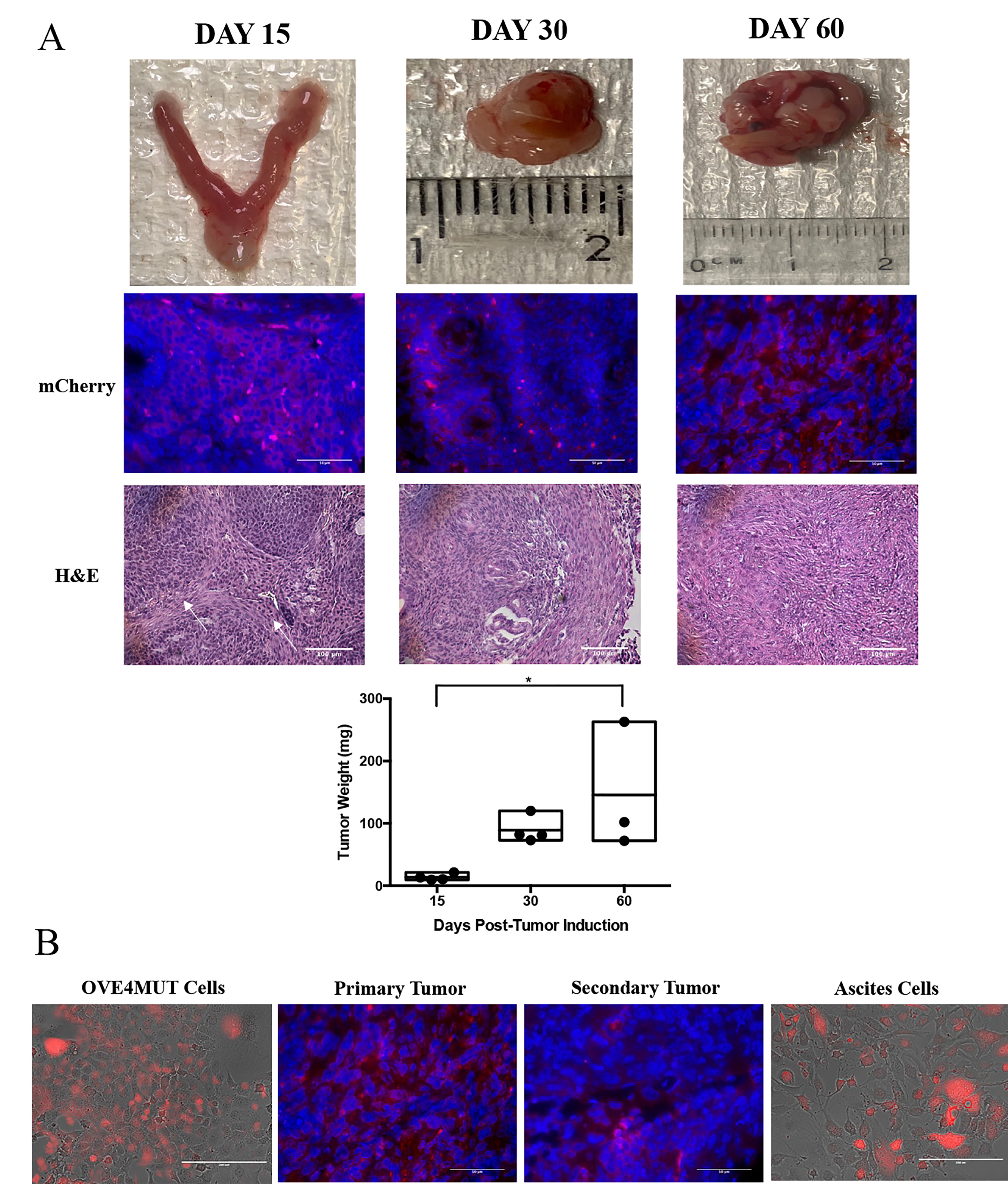

Supplement: Supplementary file 2 — Additional file 2: Supplemental Figure 2. Development of an immunocompetent, orthotopic, syngeneic murine model replicating oviductal origin of HGSC. A. mCherry-OVE4MUT cells were injected into the left oviduct of female FVB/N mice and tumor tissues were collected and weighed at 15, 30 and 60 days PTI (n = 3–4/group). Immunofluorescence staining for mCherry and H&E staining was performed on murine ovarian tumors collected at all timepoints. B. Confirmation of mCherry staining in OVE4MUT cells and aspirated ascites cells and immunofluorescence staining for mCherry on murine ovarian and secondary metastatic tumors. Images of cells were taken at 200X and images on tissues were taken at 400X. Arrows point to serous tissue in H&E staining. Bars represent mean ± SEM (*p < 0.05). Scale bars: 100 μm (H&E), 50μm (immunofluorescence-tumor); 200μm (cells). [file 13048_2023_1307_MOESM2_ESM.tif]

Western Blot Images


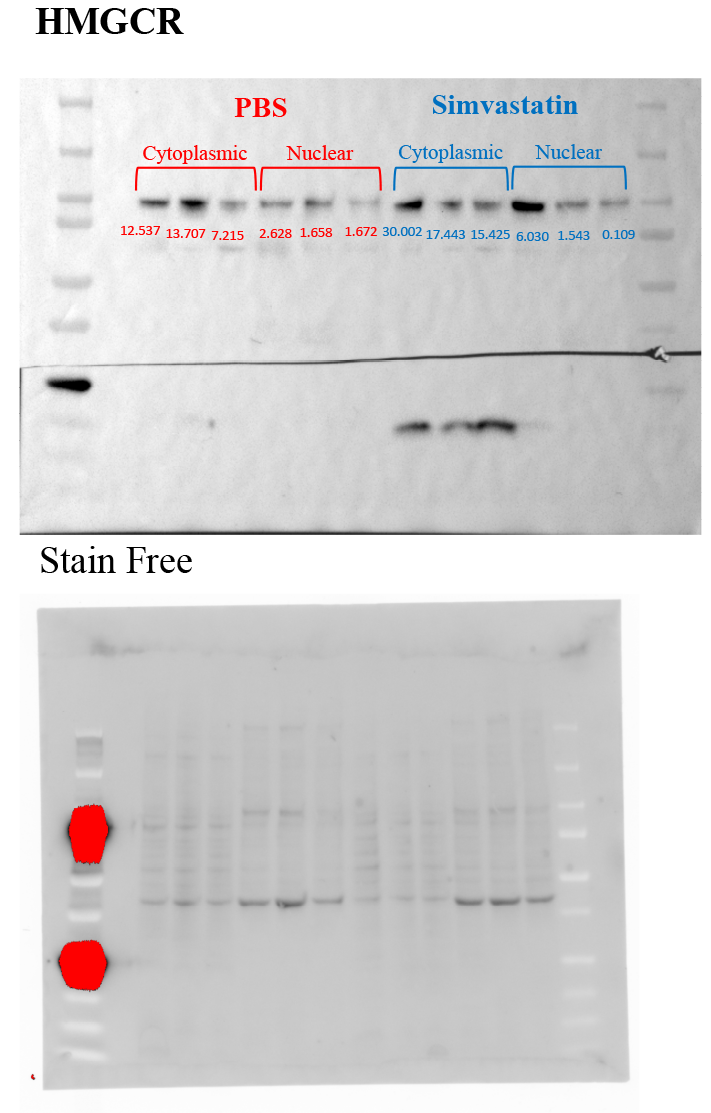


Figure 6C


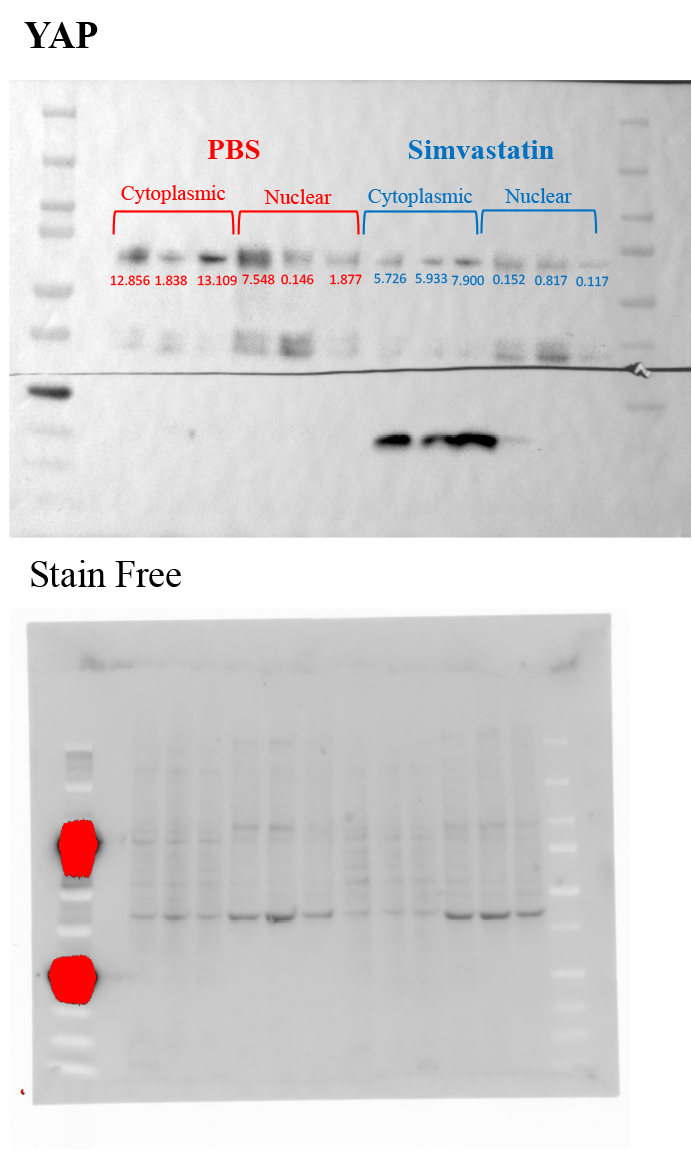


Figure 6C


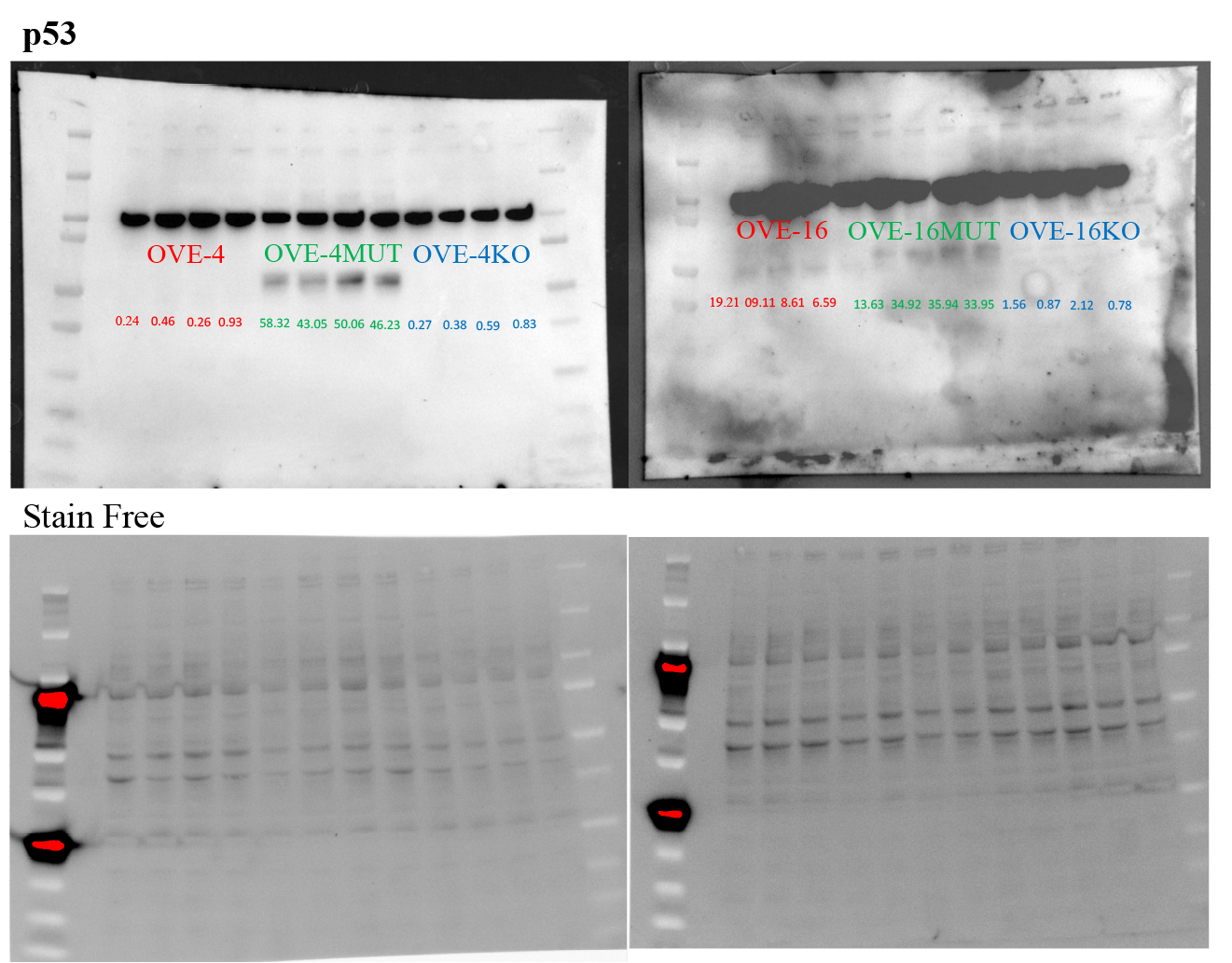


Suppl Fig 1


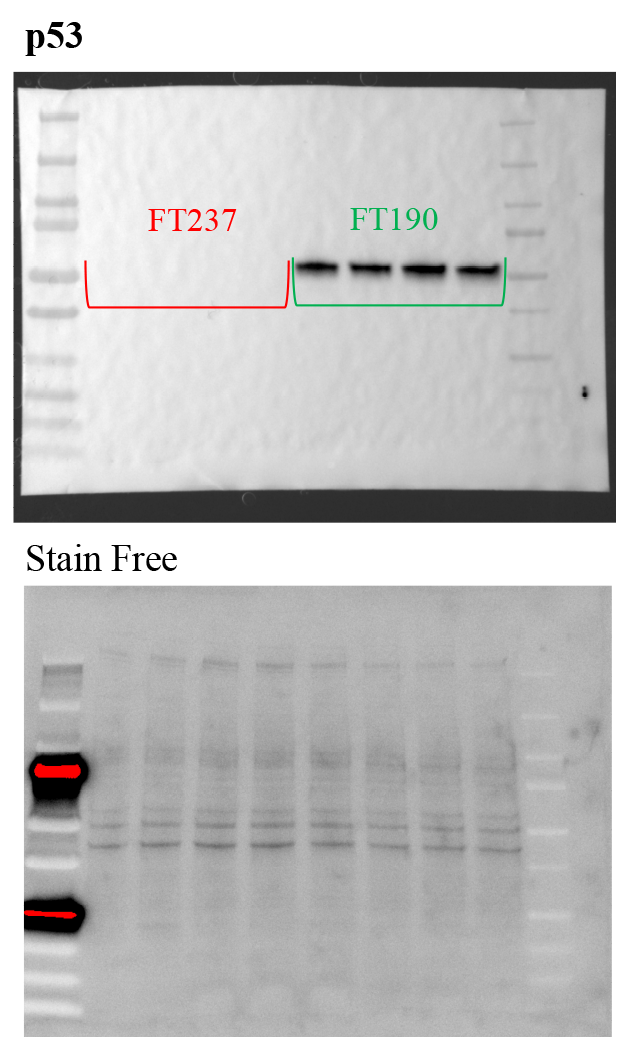


Suppl Fig 1

Supplement: Supplementary file 3 — Additional file 3. [file 13048_2023_1307_MOESM3_ESM.docx]
